# Assessing the intention to accept inquiry-based teaching pedagogy among Chinese university students: an extension of technology acceptance model

**DOI:** 10.3389/fpsyg.2024.1265047

**Published:** 2024-01-23

**Authors:** San-gen Hu, Wan-ying Wang, Xiao-Xia Wang, Ying-Mei Yin

**Affiliations:** School of Civil and Transportation Engineering, Guangdong University of Technology, Guangzhou, China

**Keywords:** inquiry-based teaching pedagogy, intention to accept, technology acceptance model, influencing factors, path analysis, university students

## Abstract

**Introduction:**

Due to the limitations of traditional didactic teaching, inquiry-based teaching has attracted increasing attention and has become an important content of curriculum teaching reform in college education. Nevertheless, it is vital to investigate students’ subjective acceptance of inquiry-based instruction and its influencing factors before inquiry-based teaching methods are widely implemented.

**Methods:**

In light of this, taking into account the psychological factors of students, an acceptance model of inquiry-based teaching pedagogy was established based on the extended technology acceptance model (TAM). Three additional variables, namely self-efficacy, implementation quality, and risk perception, were incorporated into the TAM. Firstly, subjective evaluation data of the influencing factors of inquiry teaching acceptance were obtained through a network questionnaire survey from university students in Guangdong, China, using snowball sampling and convenient sampling. A total of 485 valid questionnaires were retrieved, with an effective response rate of 88.2%. Then, internal consistency and reliability, convergent validity and discriminant validity of the model and its hypothesis were tested with reliability and validity tests. Finally, path analysis was used to examine key determinants of students’ acceptance of inquiry teaching and moderators.

**Results:**

Results indicated that the constructed model can explain the acceptability of inquiry teaching for college students by 88.6%; Attitude has a positive significant impact on behavioral intention; Perceived ease of use indirectly affects behavioral intention through perceived usefulness, while perceived usefulness indirectly affects behavioral intention through attitude; self-efficacy not only directly affects behavioral intention but also indirectly affects behavioral intention through implementation quality; implementation quality indirectly affects behavioral intention through perceived usefulness and attitude; students’ risk perception of inquiry-based teaching has no negative impact on behavioral intention.

**Conclusion:**

Overall, this study has implications for policymakers, teachers or learners in terms of the implementation and promotion of inquiry-based teaching in college classroom.

## 1 Introduction

With the development of society and the progress of science and technology, universities education is increasingly paying attention to cultivating students’ innovative thinking and problem-solving abilities (Justice et al., 2009). Traditional teaching methods are no longer able to meet the needs of students. Therefore, inquiry-based teaching methods have gradually gained widespread attention and have become an indispensable part of the curriculum teaching reform (Keselman, 2003 Kiernan and Lotter, 2019). Inquiry-based teaching is student-centered learning and teaching, which encourages students to actively discover, explore, collaborate, and communicate with their peers, in order to construct knowledge and promote the application of several problem-solving skills (Kogan and Laursen, 2014). Traditional teaching methods focus on “what to learn,” that is, what teachers teach and what students learn; while inquiry-based teaching pedagogies emphasize “how to learn” and “learning by doing” (Benlahcene et al., 2020). Besides receiving scientific knowledge directly from teachers in the classroom, students also engage in learning through inquiry and problem-solving activities. Inquiry-based teaching tries to change the focus of the classroom from “teacher’s teaching” to “student’s learning,” which can improve students’ initiative, participation, and experience in the classroom learning process unprecedently (Justice et al., 2009; Benlahcene et al., 2020). Furthermore, it can effectively enhance students’ problem-awareness, autonomous learning abilities and critical thinking skills, and teamwork spirit (Çakıroğlu and Öztürk, 2017). Inquiry-based teaching is a cutting-edge and advanced classroom teaching method vigorously advocated in the current teaching reform, and it is also an important starting point for building first-class classrooms, thus opening the “last mile” from the construction of first-class universities to the training of first-class talents. A few quantitative studies support the effectiveness of inquiry-based teaching as an instructional approach (Alfieri et al., 2011; Furtak et al., 2012; Fan and Ye, 2022).

The introduction of a new pedagogy (inquiry-based teaching) is often met with challenges and barriers within an existing program or curriculum (Justice et al., 2009). Inquiry-based teaching is a student-centered and problem-oriented teaching method that subverts traditional teaching method, and has obvious advantages in strengthening the links between teaching and research (Spronken-Smith and Walker, 2010). If the inquiry-based teaching is not accepted by students, it will be difficult to play an important role in the implementation process, and its application and popularization in college course education will also be hindered. Therefore, it is valuable to examine inquiry-based teaching acceptance from the perspective of students’ cognitive characteristics further in more detail, and identify its key influencing factors in the early stage of the application of inquiry-based teaching. Specifically, discussing students’ perspectives and their willingness to accept inquiry-based teaching will make some meaningful contributions to the sustainable development of learner-centered learning/teaching approaches, e.g., mixed methods, problem-oriented, competition-oriented (Ye et al., 2023a). Meanwhile, it has indescribable practical significance for improving its teaching effect and application promotion. For example, research results can help teachers use differentiated and student-centered inquiry-based teaching methods in different subjects.

Most research on inquiry-based teaching methods focuses on analyzing the quality of learning outcomes. To date, however, few empirically studies have explored the acceptance of inquiry-based teaching (Hao and Wang, 2019; Chen and Wang, 2020; Wang and Li, 2020; Hsu and Rowland-Goldsmith, 2021; Fan and Ye, 2022). They mainly investigated the intuitive feelings, acceptance willingness, and satisfaction of college students from different educational backgrounds on inquiry-based teaching methods by online surveys. These research results are all based on descriptive statistics and do not rely on any underlying theoretical models. Public acceptance is a crucial factor for successful technology implementation. Researchers often use the technology acceptance theory to explain public acceptance, such as the Technology Acceptance Model (TAM), the Theory of Planned Behavior (TPB), and the Unified Theory of Acceptance and Use of Technology (UTAUT), etc. The original TAM consists of four factors: perceived usefulness, perceived ease of use, attitude, and behavioral intention to use. More and more scholars extended the TAM model with adding new influencing factors (e.g., self-efficacy, and risk perception) or integrating multiple models to describe public acceptance to further enhance the predictability of the model (Gallego-Gómez et al., 2021; Vladova et al., 2021; Chou et al., 2023; Ohanu et al., 2023). The integrated model has a stronger explanatory power for acceptance willingness, which is widely regarded as the main model for studying public acceptance (Venkatesh et al., 2012). However, these models have not yet been applied to study the acceptance of inquiry-based teaching.

To sum up, research based on any single model has the problem of incomplete consideration of influencing factors, and the research results may be slightly different from the actual case. A model based on the integration of the original TAM and multiple external latent variables (influencing factors) is more explanatory and is a common practice in studying public acceptance (Ye et al., 2023b). Regrettably, current research mainly focuses on the acceptance of information technology teaching and blended teaching methods (Wu et al., 2010; Fathema et al., 2015; Teo et al., 2019; Long and Khoi, 2020; Gallego-Gómez et al., 2021; Ohanu et al., 2023), and research results cannot be fully applied to inquiry-based teaching methods. This is because the influencing factors and their paths (e.g., direct effect, indirect effect or mediating effect) to influence acceptance were different regarding different research objects. Moreover, the results of descriptive statistical analysis cannot reflect the influence mechanism. It is critical to quantify the direct effects and indirect effects of the observable psychological factors on behavioral intention. To fill this research gap, the present study proposes a novel inquiry-based teaching acceptance model that explains the subject acceptance and use of inquiry-based teaching, as well as its influencing factors, by extending TAM. Besides the predictors used in the original TAM (behavioral intention, attitudes, perceived usefulness, perceived ease of use; to be described later), external variables are important antecedents that influence learning experience. Through a review of the literature, self-efficacy, implementation quality, and risk perception are included as the external variables of the research model of acceptance of inquiry-based teaching. The reasons for adding three external variables are twofold: (1) From social cognitive theory, self-efficacy is believed to be the most relevant factors affecting human behavior in performing a specific behavior (Bandura, 1977; Venkatesh et al., 2003; Hong et al., 2019). (2) Learning satisfaction is essential for understanding how students feel about their learning experience (Hossain and Quaddus, 2012; Sockalingam, 2013). The degree of satisfaction is directly influenced by perceived learning effect and expectations of the perception (denoted as implementation quality), as well as cumulative psychological response to learning contents and learning environment (denoted as risk perception) (Yao et al., 2016).

In summary, this study aims to explore the following core research questions:

*RQ1: How do psychological factors affect students’ acceptance of inquiry-based teaching?*

*RQ2: What are the key determinants of students’ acceptance of inquiry teaching and moderators?*

## 2 Research model and hypothesis development

**Figure 1:**
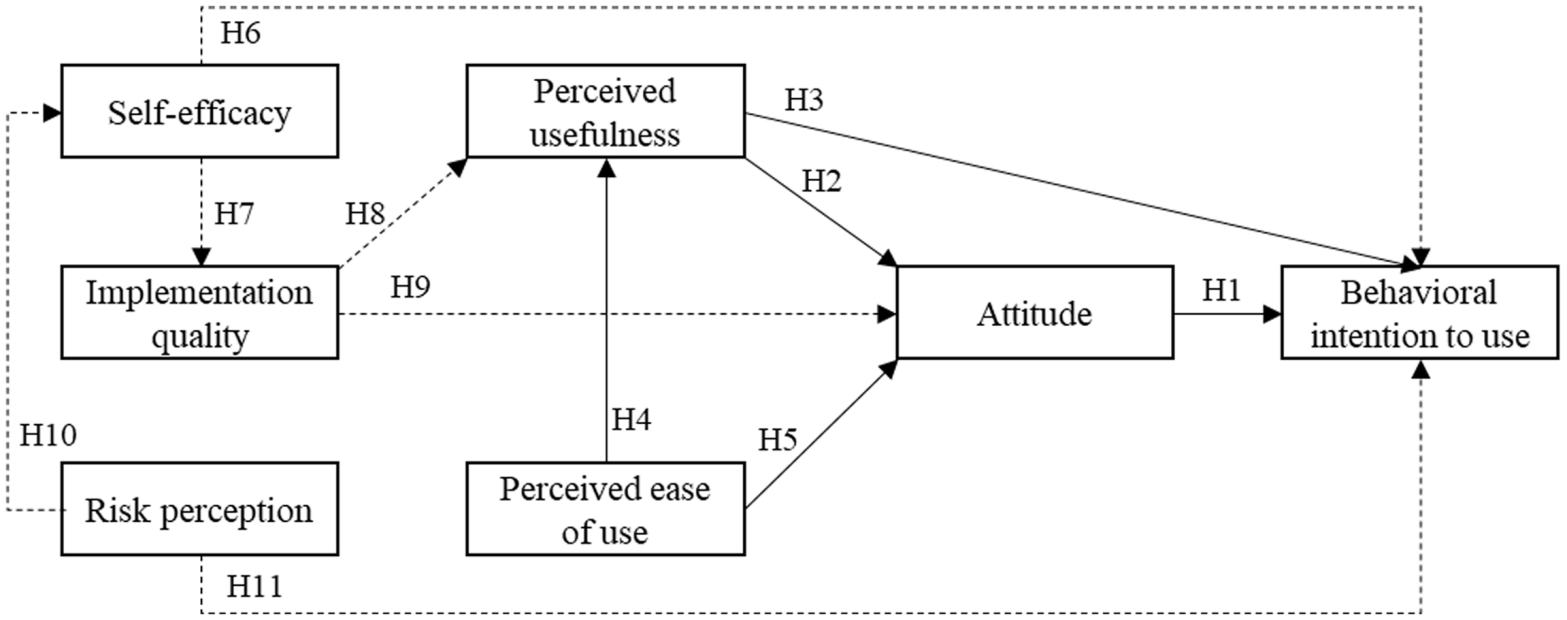
Proposed extended TAM (permission for using TAM in the current research has been obtained from the authors).

In order to provide a better understanding to the exploration of injury-based teaching acceptance among Chinese university students, three factors “self-efficacy,” “implementation quality” and “risk perception” were incorporated as external variables in the original TAM to form a new theoretical model. The proposed model (as depicted in Figure 1) was used to explore the effects of the proposed external variables on inquiry-based teaching acceptance behavior. The solid line is the path relationship of the original TAM model, and the dotted line is the path relationship of the newly added latent variable. There are two reasons for adding new latent variables: (1) Inquiry-based teaching method is an emerging teaching method that subverts traditional teaching. The perception of its teaching form, implementation quality and risk perception are key factors affecting students’ willingness to accept; (2) Self-efficacy is held to be the principal cognitive determinants of individual behavior (Wu et al., 2010). That is, students who had higher self-efficacy will have higher confidence and capability to perform a specific behavior. In the next section, brief definitions and the inferences of the proposed three factors as antecedents of injury-based teaching usage and related hypotheses are presented.

### 2.1 Attitude and behavioral intention to use

Attitude (ATT) refers to the subjective positive or negative feelings about performing the behavior (Fishbein and Ajzen, 1975; Hong et al., 2020). Here, attitude is defined as the degree to which an individual feels positive or negative about accepting injury-based teaching. Behavioral intention to use (BIU) refers to the degree to which the public uses the thing or is willing to complete a specific behavior. According to the TAM, acceptance depends on the use intention for a particular technology (Davis et al., 1989). Those who hold a positive attitude towards new technologies often have a strong behavioral intention, which ultimately affects actual use behavior (Davis et al., 1989). Ye et al. (2023b) also stated that attitudes toward technology were found to be one of the most important factors influencing the acceptance of the technology. As such, this study makes the following hypothesis:

*H1*: A positive attitude towards inquiry-based teaching will promote acceptance.

### 2.2 Perceived usefulness and perceived ease of use

In the original TAM model, perceived usefulness (PU) and perceived ease of use (PEU) are the two most fundamental factors. Perceived usefulness refers to the degree to which the public believes that use of a specific application system can improve job performance (Davis et al., 1989). Inquiry-based teaching can bring many benefits to classroom teaching, such as improving learning interest, stimulating creativity, promoting active learning and team cooperation awareness, etc. These benefits may promote students’ positive attitudes and acceptance intentions towards it. Perceived ease of use refers to the degree to which the public believes that a specific application system is easy to use (Davis et al., 1989). If students are not particularly familiar with inquiry-based teaching, they need to spend more energy to adapt and learn. Likewise, if the perceived ease of use is not good, it will have a negative impact on their acceptance of this method. Thus, this study formed theses hypotheses as suggested by Davis et al. (1989), which were corroborated by previous studies (Davis et al., 1989; Chang et al., 2015):

*H2*: Perceived usefulness will have a positive impact on the attitude towards using inquiry-based teaching.

*H3*: Perceived usefulness will have a positive impact on behavioral intention to use inquiry-based teaching.

*H4*: Perceived ease of use will have a positive impact on perceived usefulness of inquiry-based teaching.

*H5*: Perceived ease of use will have a positive impact on attitude towards using inquiry-based teaching.

### 2.3 Self-efficacy

Self-efficacy refers to an individual’s confidence in their ability to effectively complete a task or achieve a goal (Bandura, 1982; Jiang et al., 2021). According to the concept of self-efficacy, self-efficacy is defined as students’ ability and confidence to complete various tasks and challenges in the implementation process of inquiry-based teaching. In previous studies, self-efficacy was confirmed as a decisive factor in accepting and using an application system (Venkatesh et al., 2003; Wu et al., 2010). Studies also show that the improvement of self-efficacy can enhance the initiative and persistence of individual learning, thereby improving their good expectations for implementation results and behavioral intentions (Johnston et al., 2005; Francescato et al., 2006). In addition, empirical research has found that there is a significant positive correlation between self-efficacy and students’ learning attitudes and abilities (Venkatesh and Davis, 2000), that is, students with higher sense of self-efficacy are more likely to have better learning attitudes and abilities to adapt to inquiry-based teaching. They will be more recognized for its value and full of expectations for it, thereby stimulating their behavioral intentions to use this method. Following the findings of these studies, the following hypotheses were proposed:

*H6*: Self-efficacy will have a positive impact on behavioral intention to use inquiry-based teaching.

*H7*: Self-efficacy will have a positive impact on implementation quality of inquiry-based teaching.

### 2.4 Implementation quality

Implementation quality is defined as the degree of an individual’s perception of how well the system (Venkatesh and Davis, 2000). Here, implementation quality is a measure of the quality and effectiveness of inquiry-based teaching, including teacher’s process guidance, inquiry of questions, student participation and interactive atmosphere. Studies have shown that implementation quality has a significant impact on public’s positive perception of things. It is believed that implementation quality affects perceived usefulness and attitude (Venkatesh and Bala, 2008). The higher the implementation quality of inquiry-based teaching methods, the better the participation experience for students, giving them better thinking inspiration and exploration space. Therefore, they can perceive its usefulness more positively and have positive behavioral attitudes towards it (Venkatesh and Bala, 2008; Fathema et al., 2015; Teo et al., 2019). Therefore, the following hypotheses are proposed:

*H8*: Implementation quality will have a positive impact on perceived usefulness of inquiry-based teaching.

*H9*: Implementation quality will have a positive impact on attitude towards accepting inquiry-based teaching.

### 2.5 Risk perception

Risk perception (RP) refers to individuals’ risk cognition and evaluation of certain situations or behaviors, which can determine individuals’ behavior to a certain extent (Dowling and Staelin, 1994). Risk perception is introduced in this paper is that because students are not particularly familiar with inquiry-based teaching, they may exhibit negative effects such as not achieving expected teaching results or perceiving this method as having great challenges and uncertainties. Risk perception is crucial for acceptance intention (Dowling and Staelin, 1994; Lo, 2013; Alalwan et al., 2016). Risk perception is a prerequisite for trust and acceptance. Only by reducing risk perception can acceptance intention be improved (Mou, 2017). In addition, research has shown that there was a close relationship between risk perception and self-efficacy. Bandura pointed out that when individuals perceive higher risks, their sense of self-efficacy decreases (Bandura et al., 1999). Because he feels that he cannot overcome these risks and cannot successfully complete tasks or achieve goals. Conversely, when a person perceives low risks, his sense of self-efficacy increases because he believes he can easily complete a task or achieve a goal. Therefore, the following hypotheses are:

*H10*: Risk perception will have a negative impact on self-efficacy.

*H11*: Risk perception will have a negative impact on the behavioral intention of using inquiry-based teaching.

## 3 Methods

### 3.1 Participants

In total, 485 undergraduate students from universities in the Guangdong region were invited to complete the online survey on a voluntary basis. The size of the sample collected was considered sufficient and was consistent with sample size recommendations from previous research that has suggested that sample sizes should be approximately 10–20 times the model’s observed items (Schreiber et al., 2006; Nicolaou and Masoner, 2013). As the survey instrument used 23 observed items, a multiple of 20 was used to assure better survey reliability (23 × 20 = 460 < 485). All the participants were above 18 years old. Among them, 55.7% of them were male and 44.3% were female. The summary of socio-demographic of the respondents were shown in Table 1. Of the 485 students, 35.9% were sophomore and 23.5% were junior; 53% majored in Science and Technology and 23.7% majored in Social Science and Humanities. The percentage of students who often experienced inquiry-based teaching was 46.2%, and only 14.5% were “never” or “seldom” exposed to inquiry-based teaching. Invitations to participate in this study with an online survey link or quick response (QR) code from an online platform (Questionnaire Star) were sent out to 512 currently college students.

**Table 1:** Descriptive statistics of the sample.

Variable	Categorical	Percent(%)	Variable	Categorical	Percent(%)
Gender	Male	55.7	Major	Natural Science, engineering, agriculture, medicine, military	53.0
	Female	44.3		Literature, history, Philosophy	14.2
Grade	Freshman	20.4		Education, arts, science of physical culture and sports	9.5
	Sophomore	35.9		Economics, Management Science, law	22.7
	Junior	23.5		Others	0.6
	Senior	20.2	Have you been exposed to inquiry-based teaching	Never	2.5
University Rank	985 Project	5.1		Seldom	12.0
	211 Project	12.0		Occasionally	24.5
	Common	80.6		Often	46.2
	Vocational	2.3		Always	14.8

### 3.2 Measurements

As latent variables cannot be directly measured, a questionnaire was designed to collect the empirical data for this study. The purpose of the questionnaire survey is to obtain basic information about the respondents and assess their perception toward inquiry-based teaching acceptance intention and behavioral intention to accept inquiry-based teaching soon. In total, there were three sections in the questionnaire. The first section is an introduction at the beginning of the questionnaire, which provided a detailed explanation of the concept and implementation process of inquiry-based teaching methods. This introduction made sure that all respondents understood the topic well, so they could give more accurate and insightful responses. The second section collected students’ basic information, investigate demographic information such as student gender, grade, school, etc., and whether they often exposed to the use of inquiry teaching methods in classroom. The third section asked about respondents’ perception of usefulness, ease of use, attitude, and behavioral intention to use inquiry-based teaching, as well as self-efficacy, implementation quality, risk perception. The questionnaire includes 7 latent factors and a total of 23 items of measurement, all of which are developed by modifying the verified scales presented in the existing literature (see Appendix A). At the same time, these items were also appropriately modified and adjusted based a pilot test to ensure the accuracy of the questions and the reliability of the survey. All items of latent factors were asked in the Likert scale of 1–5(1 to 5: strongly disagree, disagree, neutral, agree, and strongly agree).

## 4 Research results

### 4.1 Reliability and validity test

In the factor structure, reliability and validity test are used to test the internal consistency, reliability and validity of the measured items and latent factors. The commonly used method for testing internal consistency of the measured items in the factors is Cronbach’s alpha (CA) and composite reliability (CR). When the values of CA and CR are both greater than 0.7 (Fornell and Larcker, 1981; Hatcher and O’Rourke, 2013), internal consistency and reliability are reasonable. When the values of CA and CR are both greater than 0.6 revealing poor reliability but acceptable (Huang et al., 2013; Xie et al., 2023). Factor loadings (FL) of the measured items on the factor and average variance extracted (AVE) for the factor are used to evaluate the convergent validity of the measurement model. When FL > 0.6 and AVE > 0.5 (Hair, 2009), the questionnaire results are considered to have good convergent validity. When the square root of AVE is greater than the bivariate correlation with other factors, it is considered that a factor has good discriminant validity, namely the factors in a model are empirically different from each other. The results of three tests (internal consistency, convergent validity, and discriminant validity) are shown in Tables 2 3 respectively. All values in the tables were greater than the mentioned above criteria, indicating that the proposed measurement model have good internal consistency and reliability, convergence validity and discriminant validity.

**Table 2:** Internal consistency and convergent validity test.

Factor	Item	FL	CA	AVE	CR
PEU	PEU1	0.764	0.675	0.519	0.683
	PEU2	0.675			
PU	PU1	0.789	0.776	0.536	0.776
	PU2	0.697			
	PU3	0.707			
ATT	ATT1	0.777	0.852	0.593	0.854
	ATT2	0.728			
	ATT3	0.794			
	ATT4	0.780			
RP	RP1	0.737	0.891	0.625	0.892
	RP2	0.818			
	RP3	0.742			
	RP4	0.820			
	RP5	0.829			
IQ	IQ 1	0.745	0.83	0.553	0.831
	IQ 2	0.693			
	IQ 3	0.778			
	IQ 4	0.755			
SE	SE1	0.679	0.688	0.531	0.693
	SE2	0.775			
BIU	BIU1	0.791	0.808	0.583	0.808
	BIU2	0.729			
	BIU3	0.770			

**Table 3:** Discriminant validity test.

	PEU	PU	ATT	IQ	RP	SE	BIU
PEU	**0.721**						
PU	0.390**	**0.732**					
ATT	0.417**	0.729**	**0.770**				
IQ	0.395**	0.685**	0.747**	**0.743**			
RP	−0.163**	−0.206**	−0.240**	−0.218**	**0.790**		
SE	0.460**	0.494**	0.550**	0.576**	−0.268**	**0.729**	
BIU	0.399**	0.601**	0.680**	0.670**	−0.222**	0.685**	**0.764**

**Correlation is significant at the 0.01 level.

### 4.2 Evaluation of the structural model and hypothesis testing

First, the goodness-of-fit between the proposed model and the obtained data were judged by the combination of several indices: chi-square test, comparative fit index (CFI), root mean square error of approximation (RMSEA), incremental fit index (IFI) and Tucker-Lewis’s index (TLI). A model is considered as a good fit when CMIN/DF < 3, RMSEA <0.08, CFI > 0.9, IFI > 0.9, TLI > 0.9, GFI > 0.9, and AGFI >0.8 (Baumgartner and Homburg, 1996; Hu and Bentler, 1999; Hooper et al., 2008; Kline, 2016). The numerical values of all indices and their reference standards are shown in Table 4. All indicators are within the standard range, indicating the good fit of the model.

**Table 4:** Fit indices for the tested model.

	CMIN/DF	RMSEA	CFI	IFI	TLI	GFI	AGFI
Measurement model	2.724	0.06	0.936	0.937	0.926	0.905	0.880
Recommended value	<3	<0.08	>0.9	>0.9	>0.9	>0.9	>0.8

With acceptable goodness of fit statistics, SEM was conducted to ascertain the path of relationships between the factors based on the proposed extended TAM (as shown in Figure 1). The path coefficients (standardized coefficients) and significance of each hypothesis path were calculated to test whether the hypotheses and paths of relationships between the factors in the model were supported or not. A *p*-value of 0.05 and below is accepted or supported (Roberts et al., 2012). Table 5 shows that the relationship between BIU and PU was insignificant (*p* = 0.879 > 0.05); hence, hypothesis H3, is not supported. Similarly, hypothesis H5 and H11 are also not supported.

**Table 5:** Hypothesis testing results.

Hypothesis	Path	Standardized coefficient	Unstandardized coefficient	S.E.	C.R.	*p*	Supported?
H1	BIU ← ATT	0.366	0.377	0.132	2.858	0.004	Yes
H2	ATT ← PU	0.505	0.668	0.131	5.106	***	Yes
H3	BIU ← PU	0.018	0.025	0.165	0.152	0.879	No
H4	PU ← PEU	0.194	0.120	0.04	2.977	0.003	Yes
H5	ATT ← PEU	0.053	0.043	0.033	1.312	0.190	No
H6	BIU ← SE	0.671	0.738	0.087	8.524	***	Yes
H7	IQ ← SE	0.745	0.822	0.075	10.994	***	Yes
H8	PU ← IQ	0.82	0.601	0.047	12.723	***	Yes
H9	ATT ← IQ	0.455	0.441	0.089	4.952	***	Yes
H10	SE ← RP	−0.332	−0.228	0.039	−5.829	***	Yes
H11	BIU ← RP	0.064	0.048	0.029	1.694	0.090	No

S.E. = Standardized Estimate. C.R. = Critical Ratio.

**Figure 2:**
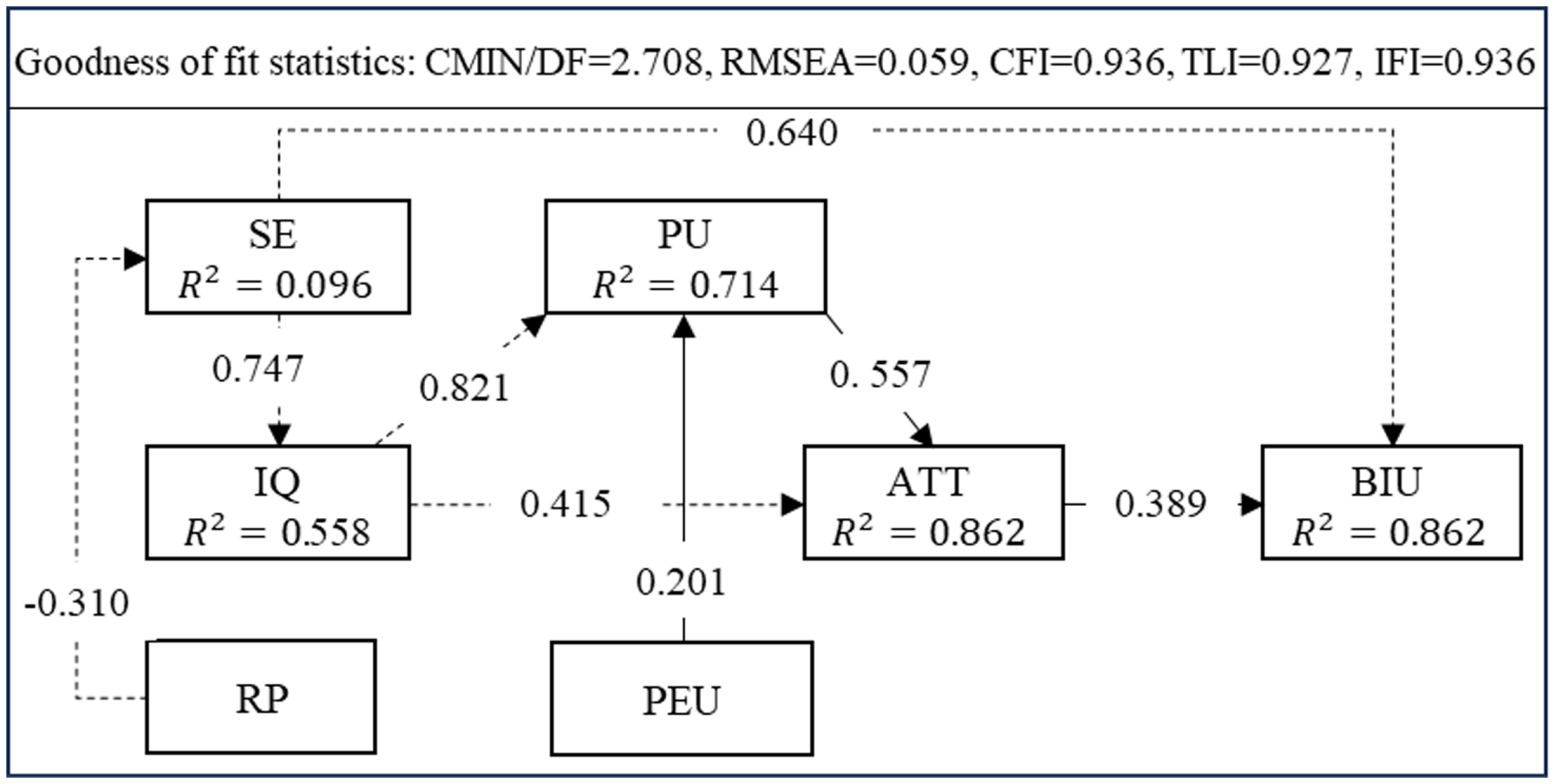
SEM results of the revised model.

Removing the above three invalid hypotheses H3, H5 and H11, the revised hypotheses regarding the paths of relationships between the factors were tested through SEM again and the results are presented in Table 6. All the paths of relationships hypothesized were found to be supported. At the same time, the fit of the revised model was re-tested. The fit indices of the revised model were CMIN/DF = 2.708, RMSEA = 0.059, CFI = 0.936, TLI = 0.927, IFI = 0.936, GFI = 0.904 and AGFI = 0.880, indicating that the fit of the revised model was also good. The results of SEM of revised model were presented in Figure 2.

**Table 6:** Revised hypothesis testing results.

Hypothesis	Path	Standardized coefficient	Unstandardized coefficient	S.E.	C.R.	*p*	Supported?
H1	BIU ← ATT	0.389	0.398	0.062	6.37	***	Yes
H2	ATT ← PU	0.557	0.751	0.118	6.382	***	Yes
H4	PU ← PEU	0.201	0.120	0.042	2.828	0.005	Yes
H6	BIU ← SE	0.640	0.704	0.081	8.676	***	Yes
H7	IQ ← SE	0.747	0.822	0.074	11.033	***	Yes
H8	PU ← IQ	0.821	0.595	0.047	12.683	***	Yes
H9	ATT ← IQ	0.415	0.405	0.079	5.145	***	Yes
H10	SE ← RP	−0.310	−0.214	0.038	−5.618	***	Yes

S.E. = Standardized Estimate. C.R. = Critical Ratio.

The technology acceptance model assumes that the use of the system is determined by BIU. There are direct and indirect effects between each latent variable and BIU in the model. The direct effect refers to the path coefficient between two latent variables, and the indirect effect refers to the product of multiple coefficients on the path, and the total effect is equal to the direct effect plus the indirect effect. Table 7 details the direct and indirect effects of several factors on BIU.

**Table 7:** Direct and indirect effects on BIU.

Variable	PEU	RP	SE	IQ	PU	ATT
Direct effect	–	–	0.640	–	–	0.389
Total indirect effect	0.044	−0.277	0.253	0.339	0.217	–
Total effect	0.044	−0.277	0.894	0.339	0.217	0.389

As shown in Table 7, only SE and ATT have the direct effect on BIU, and SE has the larger direct effect on BIU (0.64) than ATT (0.389). SE was mediated by IQ, and the magnitude of mediation was 0.253 (indirect effect on BIU). In addition, other factors only have the indirect effect on BIU. Among these factors, IQ has the largest indirect effect on BIU (0.399). PU and ATT mediated IQ with the magnitude of 0.178 and 0.161, respectively. Note that only RP have the negative indirect effect on BIU.

## 5 Discussions

Self-efficacy has the largest total effect on the acceptance of inquiry teaching methods (0.894). Self-efficacy has a direct effect on BIU (0.64), and thus can directly affect students’ acceptance of inquiry teaching. This outcome was similar to the findings of previous studies (Holden and Rada, 2011; Haynes et al., 2023). Self-efficacy also has an indirect effect on behavioral intentions through implementation quality (0.253). Self-efficacy has a significant positive effect on implementation quality (standardized path coefficient value 0.747) (Bonet, 2021). Improving self-efficacy is one of the most important ways to enhance students’ acceptance behavior intention. Therefore, when promoting and implementing inquiry-based teaching methods, the role of the lecturer should be shifted to being a facilitator and motivator who provides direction to students to find information and knowledge needed in the learning process according to students’ knowledge level, individual differences, and subject characteristics. Build and strengthen students’ confidence, enhance their sense of participation, identity and achievement, and make them more interested and confident to complete inquiry activities, so as to improve their acceptance of inquiry teaching.

Attitude (0.389) is a significant secondary determinant of students’ intentions to accept inquiry teaching methods. Attitude directly affects behavior intention, implying that the more students develop positive attitudes toward inquiry-based teaching, the more they accept it. This result consists fairly well with the literature (Akman and Turhan, 2017; Moreno et al., 2017; Nicol et al., 2022; Ye et al., 2023b), demonstrating that attitude was a significant predictor. Therefore, in order to improve students’ acceptance of inquiry-based teaching, the key lies in changing students’ understanding of inquiry-based teaching to shape positive attitude. Teachers should introduce its differences and values compared with traditional teaching methods from multiple perspectives so that students have a full understanding and recognize of its role forming a positive attitude of acceptance.

The total effect of implementation quality on the acceptance of inquiry teaching is 0.339, next to attitude. Implementation quality has no direct effect on behavioral intentions. It indirectly affects behavioral intentions through perceived usefulness and attitude. Implementation quality has a significant positive effect on perceived usefulness and attitude (standardized path coefficient values are 0.821 and 0.415), which conforms well with similar studies conducted on Edmodo acceptance (Unal and Uzun, 2021). Students were likely to believe that inquiry-based teaching is one approach to improving the effectiveness and productivity for learning by moving toward more student-directed, interactive methods of learning and this might have influenced their perception of usefulness and attitude (Justice et al., 2009). Therefore, in the implementation of inquiry teaching, teachers should play the role of guide and tutor, provide necessary guidance and support to students in the exploration process; It is important to help students solve problems and answer questions to improve their sense of participation and confidence in learning on their own, and to ensure that each student can achieve the established learning objectives and improve students’ perception of implementation quality. If students believe that inquiry teaching are meaningful and useful for learning, they will perceive its usefulness for learning and generating positive behavioral attitudes.

The total effect of perceived ease of use and perceived usefulness on the acceptance intention of inquiry teaching is 0.044 and 0.217, respectively. Consistent with many other research (Davis et al., 1989; Moon and Kim, 2001), this result indicates that, although ease of use is clearly important, and the usefulness of inquiry-based teaching methods is even more important and should be emphasized. It is worth noting that perceived usefulness has no direct effect on behavioral intentions but indirectly affects behavioral intentions through attitudes, which is inconsistent with prior findings. This implies that students’ judgment of the usefulness of inquiry-based teaching will significantly affect their cognitive aspects of attitudes towards it and thus play an important role in their acceptance behavior. Contrary to the presupposition, perceived ease of use has no significant effect on attitude, but only indirectly affects attitude through perceived usefulness. A possible explanation is that first students evaluate how easy or difficult it is to learn with the inquiry-based teaching, then they look at the usefulness of it for them. If they find it as an ‘useful’ method for them then they develop a positive attitude towards it. The positive attitudes lead them to develop a positive intention to accept it. In other words, if they find it as an ‘unprofitable’ method for them, they may hold a negative attitude towards it.

The total effect of risk perception on the acceptance intention of inquiry teaching methods is −0.277, which is the only factor negatively correlated with behavioral intention, indicating that students with lower risk perception are more they are more likely to be motivated to accept inquiry-based teaching or vice versa. This result was in line with the literature (Zhang et al., 2019). Risk perception has no direct effect on behavioral intentions, but only indirectly affects behavioral intentions through self-efficacy. Risk perception has a significant negative impact on self-efficacy. Therefore, teachers should pay attention to process guidance, question design and inquiry atmosphere when using inquiry-based teaching method. Specifically, teachers need to give continuous guidance and guidance to students, so that students can find the right direction of inquiry; Open questions should be designed according to the content of teaching materials and teaching objectives, and each student should be guided to solve the problems through experiments, discussions, observations, etc. Some ice-breaking activities can also be used to ease the classroom atmosphere, cultivate students’ interest and willingness to explore, encourage students to share and cooperate, and allow each student to complete the exploration activities and knowledge learning in a pleasant environment.

## 6 Conclusion and implications

### 6.1 Conclusion

This study assesses the acceptability of inquiry-based teaching at the subjective level of students in higher education. Theoretically, this paper constructs a complex model that helps to understand the factors influencing the acceptance of inquiry-based teaching methods. The whole extended TAM accounted for 88.6% of the variance in explaining students’ behavioral intention to accept inquiry-based teaching, indicating its good applicability to understand the acceptance of inquiry teaching methods. The extended TAM was again empirically validated as a theoretical model for future research on the use of new teaching/learning methods in educational settings.

The research results showed that, all latent variables have a significant impact on the behavioral intention to accept inquiry-based teaching: Self-efficacy (0.894), Attitude (0.389), Implementation quality (0.339), Perceived Usefulness (0.217), Perceived Ease of Use (0.044), and Perceived Risk (−0.277). Self-efficacy and attitude directly influence the intention to accept, while other factors have an indirect impact. Specifically, self-efficacy had a positive impact on implementation quality and behavioral intention to use; implementation quality had a positive impact on perceived usefulness and attitude; perceived risk had a negative impact on self-efficacy; perceived ease of use had a positive impact on perceived usefulness; perceived usefulness had a positive effect on attitude, while attitude had a positive effect on behavioral intention to use.

### 6.2 Practical implications

In terms of practical application, these details of the link between inquiry-based teaching and various factors provided theoretical guidance and practical support to improve the application of inquiry-based teaching in college education and the quality of inquiry-based instruction. First of all, the study results revealed that self-efficacy was the most critical and salient factor in determining users’ acceptance of inquiry-based teaching. This can be achieved by reducing students’ worries, anxieties and fears when they engaged in a new teaching method, given that perceived risk was found to be negatively related to students’ level of self-efficacy. Therefore, it is important to inform students about the features, usefulness of it so that they can gain an in-depth understanding of the features of inquiry-based teaching method and become more confident in achieving desired tasks. At the same time, teachers should fully understand the content of the textbook, design inquiry questions around teaching goals and knowledge abilities, so that students can learn knowledge points and internalize them through the exploration and research of the problems, enhance the interaction and assistance between teachers and students and students, and stimulate every student’s confidence, interest and cooperation in exploration. Secondly, students’ positive attitude should be set up in the process of popularization of inquiry-based teaching, and efforts should be made to improve the implementation quality. For students who hold negative attitudes or are dissatisfied with implementation quality, lecturers should pay attention to their inner needs and value orientation in time, and focus on giving help and encouragement; Finally, teachers should improve their ability to teach, the adaption of situations and expertise and skill to the gain the student performance (Alharbi, 2022). They should have practical knowledge in multiple aspects, such as interpersonal knowledge and strategic knowledge. Teachers can design inquiry questions according to classroom teaching content and teaching materials, carefully arrange tasks, guide and supervise processes and evaluate inquiry results. The entire inquiry process is student-centered, respecting their individuality to achieve their self-growth, so that students can perceive the practical value and usefulness of inquiry-based teaching methods and thus reduce their risk perception.

### 6.3 Contributions

First, on the authors’ knowledge, this is the first adoption of TAM to model the acceptance behavior of inquiry-based teaching methods. This is also the first time that self-efficacy was identified as the most critical antecedent in exploring determinants of students’ acceptance for inquiry-based teaching methods, and directly influenced the intention to accept. This finding is not in line with previous studies, which have reported that the role of self-efficacy was supplementary or mediated by other factors (Wu et al., 2010; Unal and Uzun, 2021), probably due to limitations in the proposed models or the survey population.

The second theoretical contribution is that this study results help clarifying the conflicting results on the importance of perceived risk to BIU in the previous literature (Martins et al., 2014; Mou et al., 2015; Alalwan et al., 2016). Our results revealed that perceived risk would not directly determine students’ behavioral intention towards inquiry-based teaching methods but would affect it indirectly by influencing students’ level of self-efficacy.

The third theoretical contribution argues that, this study integrated the classic technology acceptance model theory, learners’ cognitive theory (self-efficacy and implementation quality), and risk perception theory (perceived risk) to investigate students’ acceptance of inquiry-based teaching. For inquiry-based teaching, students’ role in student-centered teaching approach is significantly different from their role in teacher-centered learning approach (Hannafin and Land, 1997). Therefore, it is important to identify perceptions of students about teaching and learning processes, perceptions regarding student-centered learning contexts, and their belief in one’s capability to perform certain learning tasks, which can be well identified and elucidated by these factors in the proposed model.

### 6.4 Limitations of this study and future work

The results of our study need to be interpreted in consideration of several limitations. One limitation is that the minority of the respondents have not actually been exposed to the inquiry-based teaching methods and have little or no understanding about it. The level of self-efficacy, attitude and perceived risk as well as their antecedents would change in the future, with students’ more familiarity with the approach. Another limitation is that subjective measure of latent variables might not represent objective behaviors. Moreover, due to the limited number of questionnaires, research results cannot reflect the acceptance intention of all university students. In the future, it is possible to consider further expanding the region of investigation and sample size, especially increasing target student groups who have actual experience. Longitudinal studies are necessary to further clarify how key determinants (i.e., self-efficacy, attitude) and their role in student acceptance evolve after students have more interacting experience with inquiry-based teaching methods.

## Data availability statement

The raw data supporting the conclusions of this article will be made available by the authors, without undue reservation.

## Ethics statement

The studies involving humans were approved by Ethics Board of the School of Civil and Transportation Engineering, Guangdong University of Technology. The studies were conducted in accordance with the local legislation and institutional requirements. The participants provided their written informed consent to participate in this study. Written informed consent was obtained from the individual(s) for the publication of any potentially identifiable images or data included in this article.

## Author contributions

S-gH: Conceptualization, Methodology, Writing – original draft, Writing – review & editing. W-yW: Funding acquisition, Methodology, Validation, Writing – review & editing. X-XW: Conceptualization, Funding acquisition, Investigation, Supervision, Validation, Writing – review & editing. Y-MY: Investigation, Writing – review & editing.

## APPENDIX A Latent factors and survey items.

**Table:**

Factors	Items	Description of each item	Sources (modified from)
Perceived ease of use (PEU)	PEU1	It is easy for me to conduct inquiry activities according to the questions raised by the teacher.	Davis (1989)
	PEU2	The implementation process of inquiry teaching is simple and easy to understand.	
Perceived usefulness(PU)	PU1	Inquiry-based teaching can enhance my interest and awareness of learning.	Davis (1989)
	PU2	Inquiry-based teaching can improve my learning initiative and research ability.	
	PU3	Inquiry-based teaching can deepen my understanding and application of knowledge and greatly improve my learning effect.	
Attitude (ATT)	ATT1	I think it is worthwhile to use inquiry-based teaching in classroom.	Long and Khoi (2020)
	ATT2	I hope the inquiry-based teaching will be adopted in the classroom.	
	ATT3	I find the inquiry-based teaching attractive in the classroom.	
	ATT4	I find the inquiry-based approach to classroom teaching very enjoyable.	
Implementation Quality (IQ)	IQ1	I think the teacher’s guidance and enlightenment to students in the process of inquiry-based teaching is very good.	Davis (1989)
	IQ2	I think the questions raised in the inquiry-based classroom are very probing and enlightening.	
	IQ3	I think the inquiry-based teaching has had a very good participation experience in the classroom.	
	IQ4	I think the teacher-student interaction and group collaboration in the inquiry-based teaching classroom is very good.	
Risk Perceived(RP)	RP1	I am worried that the teacher’s guidance is not timely, and the whole class will become loose, feeling like “shepherding sheep.”	This study
	RP2	I am afraid that the questions of inquiry are too difficult for me to achieve my learning goals.	
	RP3	Interacting with teachers, discussing, and collaborating with group members makes me shy, irritable and anxious.	
	RP4	With the inquiry-based teaching, I am afraid that the knowledge received is not systematic, but fragmented and scattered	
	RP5	I am worried that I will not be able to complete the learning task of independent inquiry well because of my lack of consciousness or enthusiasm	
Self-efficacy (SE)	SE1	I can easily acquire useful knowledge for myself in inquiry teaching activities	This study
	SE2	I enjoy interacting with teachers and working collaboratively with team members to complete tasks	
Behavioral intention to use (BIU)	BIU1	I will actively recommend inquiry-based teaching courses to my classmates around me	Davis (1989)	BIU2	I would invest more time and energy in inquiry-based classes	BIU3	I am willing to participate in practical tasks or experiments in the inquiry-based classroom

## References

[ref1] Akman I. Turhan C. (2017). User acceptance of social learning systems in higher education: an application of the extended technology acceptance model. Innov. Educ. Teach. Int. 54, 229–237. doi: 10.1080/14703297.2015.1093426

[ref2] Alalwan A. A. Dwivedi Y. K. Rana N. P. Williams M. D. (2016). Consumer adoption of mobile banking in Jordan: examining the role of usefulness, ease of use, perceived risk and self-efficacy. J. Enterp. Inf. Manag. 29, 118–139. doi: 10.1108/JEIM-04-2015-0035

[ref3] Alfieri L. Brooks P. J. Aldrich N. J. Tenenbaum H. R. (2011). Does discovery-based instruction enhance learning? J. Educ. Psychol. 103, 1–18. doi: 10.1037/a0021017

[ref4] Alharbi A. A. (2022). Is the emergency distance teaching experience different in postgraduate programs?: students’ voices. Int. J. Online Pedagog. Course Des. 12, 1–16. doi: 10.4018/IJOPCD.302084

[ref5] Bandura A. (1977). Self-efficacy: toward a unifying theory of behavioral change. Psychol. Rev. 84, 191–215. doi: 10.1037/0033-295X.84.2.191, PMID: 847061

[ref6] Bandura A. (1982). Self-efficacy mechanism in human agency[J]. Am. Psychol. 37, 122–147. doi: 10.1037/0003-066X.37.2.122

[ref7] Bandura A. Freeman W. H. Lightsey R. (1999). Self-efficacy: the exercise of control [J]. J. Cogn. Psychother. 13, 158–166. doi: 10.1891/0889-8391.13.2.158

[ref8] Baumgartner H. Homburg C. (1996). Applications of structural equation modeling in marketing and consumer research: a review. Int. J. Res. Mark. 13, 139–161. doi: 10.1016/0167-8116(95)00038-0

[ref9] Benlahcene A. Lashari S. A. Lashari T. A. Shehzad M. W. Deli W. (2020). Exploring the perception of students using student-centered learning approach in a Malaysian public university. Int. J. Higher Educ. 9, 204–217. doi: 10.5430/ijhe.v9n1p204

[ref10] Bonet B. (2021). Exploring middle school science teachers’ self-efficacy with inquiry-based learning: A case study. Doctoral dissertation, Northcentral University.

[ref11] Çakıroğlu Ü. Öztürk M. (2017). Flipped classroom with problem based activities: exploring self-regulated learning in a programming language course. Int. Forum Educ. Technol. Society 20, 337–349. doi: 10.1109/ICCCNT.2016.7568515

[ref12] Chang C. C. Hung S. W. Cheng M. J. Wu C. Y. (2015). Exploring the intention to continue using social networking sites: the case of Facebook. Technol. Forecast. Soc. Chang. 95, 48–56. doi: 10.1016/j.techfore.2014.03.012

[ref13] Chen Y. Wang Y. (2020). A study on student acceptance of inquiry-based learning approach in a blended learning environment. J. Educ. Technol. Develop. Exchange 13, 1–14.

[ref14] Chou S. F. Horng J. S. Liu C. H. Lin J. Y. Chen L. (2023). Discovering the processes of undergraduate hospitality students’ acceptance of facebook teaching interventions. Educ. Inf. Technol. 28, 15245–15265. doi: 10.1007/s10639-023-11836-z PMC10136397 37361807

[ref15] Davis F. D. (1989). Perceived usefulness, perceived ease of use, and user acceptance of information technology. MIS Q. 13, 319–340. doi: 10.2307/249008

[ref16] Davis F. D. Bagozzi R. P. Warshaw P. R. (1989). User acceptance of computer technology: a comparison of two theoretical models. Manag. Sci. 35, 982–1003. doi: 10.1287/mnsc.35.8.982

[ref17] Dowling G. R. Staelin R. (1994). A model of perceived risk and intended risk-handling activity. J. Consum. Res. 21, 119–134. doi: 10.1086/209386

[ref18] Fan J. Y. Ye J. H. (2022). The effectiveness of inquiry and practice during project design courses at a technology university. Front. Psychol. 13:859164. doi: 10.3389/fpsyg.2022.859164 35664202 PMC9158470

[ref19] Fathema N. Shannon D. Ross M. (2015). Expanding the technology acceptance model (TAM) to examine faculty use of learning management systems (LMSs) in higher education institutions. J. Online Learn. Teach. 11, 210–232. doi: 10.1080/08923647.2015.1029183

[ref20] Fishbein M. Ajzen I. (1975). Belief, attitude, intension and behavior: An introduction to theory and research, Reading, MA: Addison Wesley.

[ref9002] Fornell C. Larcker D. F. (1981). Evaluating structural equation models with unobservable variables and measurement error. J. Mark. Res. 18, 39–50. doi: 10.1177/002224378101800104

[ref21] Francescato D. Porcelli R. Mebane M. Cuddetta M. Klobas J. Renzi P. (2006). Evaluation of the efficacy of collaborative learning in face-to-face and computer-supported university contexts. Comput. Hum. Behav. 22, 163–176. doi: 10.1016/j.chb.2005.03.001

[ref22] Furtak E. M. Seidel T. Iverson H. Briggs D. C. (2012). Experimental and quasi-experimental studies of inquiry-based science teaching: a meta-analysis. Rev. Educ. Res. 82, 300–329. doi: 10.3102/0034654312457206

[ref23] Gallego-Gómez C. De-Pablos-Heredero C. Montes-Botella J. L. (2021). Change of processes in the COVID-19 scenario: from face-to-face to remote teaching-learning systems. Sustainability 13:10513. doi: 10.3390/su131910513

[ref9004] Hair J. (2009). Multivariate data analysis. Faculty Publications. Available at: https://digitalcommons.kennesaw.edu/facpubs/2925

[ref24] Hannafin M. J. Land S. M. (1997). The foundations and assumptions of technology-enhanced student-centered learning environments. Instr. Sci. 25, 167–202. doi: 10.1023/A:1002997414652

[ref25] Hao L. Wang D. (2019). Exploratory learning in higher education: students’ perceptions and implications for curriculum design. Front. Psychol. 10:2042.31551880

[ref9003] Hatcher L. O’Rourke N. (2013). A step-by-step approach to using SAS for factor analysis and structural equation modeling. Sas Institute.

[ref26] Haynes M. Brown A. Nichols K. Parveen Musofer R. (2023). Measurement of student attitudes to science and association with inquiry-based learning in regional schools. Int. J. Sci. Educ. 45, 593–612. doi: 10.1080/09500693.2023.2168138

[ref9006] Holden H. Rada R. (2011). Understanding the influence of perceived usability and technology self-efficacy on teachers’ technology acceptance. J. Res. Technol. Educ. 43, 343–367.

[ref27] Hong J. C. Chen M. L. Ye J. H. (2020). Acceptance of YouTube applied to dance learning. Int. J. Inform. Educ. Technol. 10, 7–13. doi: 10.18178/ijiet.2020.10.1.1331

[ref28] Hong J. C. Ye J. H. Fan J. Y. (2019). STEM in fashion design: the roles of creative self-efficacy and epistemic curiosity in creative performance. Eurasia J. Mathematics Sci. Technol. Educ. 15, 1–18. doi: 10.29333/ejmste/108455

[ref29] Hooper D. Coughlan J. Mullen M. R. (2008). Structural equation modelling: Guidelines for determining model fit. Electron. J. Bus. Res. Methods 6, 53–60. doi: 10.21427/D7CF7R

[ref31] Hossain M. A. Quaddus M. (2012). Expectation–confirmation theory in information system research: a review and analysis. Inform. Syst. Theory 28, 441–469. doi: 10.1007/978-1-4419-6108-2_21

[ref32] Hsu J. L. Rowland-Goldsmith M. (2021). Student perceptions of an inquiry-based molecular biology lecture and lab following a mid-semester transition to online teaching. Biochem. Mol. Biol. Educ. 49, 15–25. doi: 10.1002/bmb.21478, PMID: 33301654

[ref33] Hu L. T. Bentler P. M. (1999). Cutoff criteria for fit indexes in covariance structure analysis: conventional criteria versus new alternatives. Struct. Equ. Model. 6, 1–55. doi: 10.1080/10705519909540118

[ref34] Huang C. C. Wang Y. M. Wu T. W. Wang P. A. (2013). An empirical analysis of the antecedents and performance consequences of using the Moodle platform. Int. J. Inform. Educ. Technol. 3, 217–221. doi: 10.7763/IJIET.2013.V3.267

[ref35] Jiang M. Y. C. Jong M. S. Y. Lau W. W. F. Meng Y. L. Chai C. S. Chen M. (2021). Validating the general extended technology acceptance model for e-learning: evidence from an online English as a foreign language course amid COVID-19. Front. Psychol. 12:671615. doi: 10.3389/fpsyg.2021.671615, PMID: 34658995 PMC8517242

[ref36] Johnston J. Killion J. Oomen J. (2005). Student satisfaction in the virtual classroom. Internet J. Allied Health Sci. Pract. 3:6. doi: 10.46743/1540-580X/2005.1071

[ref37] Justice C. Rice J. Roy D. Hudspith B. Jenkins H. (2009). Inquiry-based learning in higher education: administrators’ perspectives on integrating inquiry pedagogy into the curriculum. High. Educ. 58, 841–855. doi: 10.1007/s10734-009-9228-7

[ref38] Keselman A. (2003). Supporting inquiry learning by promoting normative understanding of multivariable causality. J. Res. Sci. Teach. 40, 898–921. doi: 10.1002/tea.10115

[ref39] Kiernan D. A. Lotter C. (2019). Inquiry-based teaching in the college classroom: the nontraditional student. Am. Biol. Teach. 81, 479–484. doi: 10.1525/abt.2019.81.7.479

[ref40] Kline R. B. (2016). Principles and practice of structural equation modeling. 4th ed. Guilford publications.

[ref41] Kogan M. Laursen S. L. (2014). Assessing long-term effects of inquiry-based learning: a case study from college mathematics. Innov. High. Educ. 39, 183–199. doi: 10.1007/s10755-013-9269-9

[ref42] Lo A. Y. (2013). The role of social norms in climate adaptation: mediating risk perception and flood insurance purchase. Glob. Environ. Chang. 23, 1249–1257. doi: 10.1016/j.gloenvcha.2013.07.019

[ref43] Long N. Khoi B. (2020). The intention to study using zoom during the SARS-CoV-2 pandemic. Int. J. Emerg. Technol. Learn. 15, 195–216. doi: 10.3991/ijet.v15i21.16777

[ref44] Martins C. Oliveira T. Popovič A. (2014). Understanding the internet banking adoption: a unified theory of acceptance and use of technology and perceived risk application. Int. J. Inf. Manag. 34, 1–13. doi: 10.1016/j.ijinfomgt.2013.06.002

[ref9001] Mou Y. (2017). The research of public acceptance towards nuclear power in China based on the dual-factor model of risk perception. Energy Procedia. 105, 975–980. doi: 10.1016/j.egypro.2017.03.367

[ref45] Moon J. W. Kim Y. G. (2001). Extending the TAM for a world-wide-web context. Inf. Manag. 38, 217–230. doi: 10.1016/S0378-7206(00)00061-6

[ref46] Moreno V. Cavazotte F. Alves I. (2017). Explaining university students’ effective use of e-learning platforms. Br. J. Educ. Technol. 48, 995–1009. doi: 10.1111/bjet.12469

[ref47] Mou J. Shin D. H. Cohen J. F. (2015). Trust and risk in consumer acceptance of e-services. Electron. Commer. Res. 17, 255–288. doi: 10.1007/s10660-015-9205-4

[ref48] Nicol C. B. Gakuba E. Habinshuti G. (2022). Effects of inquiry-based chemistry experimentation on Students' attitudes towards the teaching and learning of chemistry. J. Balt. Sci. Educ. 21, 663–679. doi: 10.33225/jbse/22.21.663

[ref49] Nicolaou A. I. Masoner M. M. (2013). Sample size requirements in structural equation models under standard conditions. Int. J. Account. Inf. Syst. 14, 256–274. doi: 10.1016/j.accinf.2013.11.001

[ref50] Ohanu I. B. Shodipe T. O. Ohanu C. M. Anene-Okeakwa J. E. (2023). System quality, technology acceptance model and theory of planned behaviour models: agents for adopting blended learning tools. E Learn. Digital Media 20, 255–281. doi: 10.1177/20427530221108031

[ref9005] Roberts S. C. Ghazizadeh M. Lee J. D. (2012). Warn me now or inform me later: Drivers acceptance of real-time and post-drive distraction mitigation systems. Int. J. Hum. Comput. Stud. 70, 967–979. doi: 10.1016/j.ijhcs.2012.08.002

[ref51] Schreiber J. B. Nora A. Stage F. K. Barlow E. A. King J. (2006). Reporting structural equation modeling and confirmatory factor analysis results: a review. J. Educ. Res. 99, 323–338. doi: 10.3200/JOER.99.6.323-338

[ref52] Sockalingam N. (2013). The relation between student satisfaction and student performance in blended learning curricula. Int. J. Learn. Annu. Rev. 18, 121–134. doi: 10.18848/1447-9494/cgp/v18i12/47842

[ref53] Spronken-Smith R. Walker R. (2010). Can inquiry-based learning strengthen the links between teaching and disciplinary research? Stud. High. Educ. 35, 723–740. doi: 10.1080/03075070903315502

[ref54] Teo T. Zhou M. Fan A. C. W. Huang F. (2019). Factors that influence university students’ intention to use Moodle: a study in Macau. Educ. Technol. Res. Dev. 67, 749–766. doi: 10.1007/s11423-019-09650-x

[ref55] Unal E. Uzun A. M. (2021). Understanding university students’ behavioral intention to use Edmodo through the lens of an extended technology acceptance model. Br. J. Educ. Technol. 52, 619–637. doi: 10.1111/bjet.13046

[ref56] Venkatesh V. Bala H. (2008). Technology acceptance model 3 and a research agenda on interventions. Decis. Sci. 39, 273–315. doi: 10.1111/j.1540-5915.2008.00192.x

[ref57] Venkatesh V. Davis F. D. (2000). A theoretical extension of the technology acceptance model: four longitudinal field studies. Manag. Sci. 46, 186–204. doi: 10.1287/mnsc.46.2.186.11926

[ref58] Venkatesh V. Morris M. G. Davis G. B. Davis F. D. (2003). User acceptance of information technology: toward a unified view. MIS Q. 27, 425–478. doi: 10.2307/30036540

[ref59] Venkatesh V. Thong J. Y. Xu X. (2012). Consumer acceptance and use of information technology: extending the unified theory of acceptance and use of technology. MIS Q. 36, 157–178. doi: 10.2307/41410412

[ref60] Vladova G. Ullrich A. Bender B. Gronau N. (2021). Students’ acceptance of technology-mediated teaching-how it was influenced during the COVID-19 pandemic in 2020: a study from Germany. Front. Psychol. 12:636086. doi: 10.3389/fpsyg.2021.636086, PMID: 33613405 PMC7887425

[ref61] Wang B. Li Y. (2020). Evaluating the impact of inquiry-based learning on student engagement and learning outcomes: an online survey study. J. Educ. Learn. 9, 152–165.

[ref62] Wu J. H. Tennyson R. D. Hsia T. L. (2010). A study of student satisfaction in a blended e-learning system environment. Comput. Educ. 55, 155–164. doi: 10.1016/j.compedu.2009.12.012

[ref63] Xie Q. Liu X. Fan L. Peng S. Zeng Y. (2023). Evaluation of equivalent crack propagation length and fracture energy of two commonly used rock fracture toughness test configurations based on Bažant’s size effect law. Eng. Fract. Mech. 281:109067. doi: 10.1016/j.engfracmech.2023.109067

[ref64] Yao J. Yang T. Ding X. Chen X. W. (2016). The concept tracing, concept definition and implication analysis of urban community sports’ public service’s satisfaction. J. Xi’an Phys. Educ. University 33, 48–56.

[ref65] Ye J. H. Chen M. Y. Hao Y. W. (2023a). Editorial: teaching and learning in higher education: the role of emotion and cognition. Front. Psychol. 14:1230472. doi: 10.3389/fpsyg.2023.1230472, PMID: 37469887 PMC10352986

[ref66] Ye J. H. Lee Y. S. Wang C. L. Nong W. Ye J.-N. Sun Y. (2023b). The continuous use intention for online learning of Chinese vocational students in the post-epidemic era: the extended technology acceptance model and expectation confirmation theory. Sustainability 15:1819. doi: 10.3390/su15031819

[ref67] Zhang T. Tao D. Qu X. Zhang X. Lin R. Zhang W. (2019). The roles of initial trust and perceived risk in public’s acceptance of automated vehicles. Transport. Res. Part C 98, 207–220. doi: 10.1016/j.trc.2018.11.018

